# Antibody response to enterotoxigenic *Bacteroides fragilis* of Filipino colorectal cancer patients

**DOI:** 10.2478/abm-2023-0070

**Published:** 2023-12-28

**Authors:** Ana Maria D. Cariño, Gregg Austine Balanag, Edrienne Myenna Magat, Allan Fellizar, Teresa Sy Ortin, Oliver Villaflores, Leonardo Guevarra, Pia Marie Albano

**Affiliations:** Research Center for the Natural and Applied Sciences, University of Santo Tomas, España Manila 1015, Philippines; The Graduate School, University of Santo Tomas, España Manila 1015, Philippines; College of Teacher Education, Quirino State University, Quirino 3401, Philippines; Hematology Division, Mariano Marcos Memorial Hospital and Medical Center, Batac, Ilocos Norte 2906, Philippines; Benavides Cancer Institute, University of Santo Tomas Hospital, España Manila 1015, Philippines; Department of Biochemistry, Faculty of Pharmacy, University of Santo Tomas, España Manila 1015, Philippines; Department of Biological Sciences, College of Science, University of Santo Tomas, España Manila 1015, Philippines

**Keywords:** antibodies, colorectal cancer, ELISA, enterotoxigenic *Bacteroides fragilis*, gut microbiota

## Abstract

**Background:**

Several species of the gut microbiota have been implicated in colorectal cancer (CRC) development. The anaerobic bacterium enterotoxigenic *Bacteroides fragilis* (ETBF), has been identified to produce fragilysin, a toxin known to cleave E-cadherin, thereby leading to carcinogenesis.

**Objective:**

To determine the antibody response of CRC patients against ETBF to ascertain whether significant difference exists or whether antibody response is related to tumor grade and tumor stage.

**Methods:**

Informed consent was obtained from histologically confirmed CRC casesand their age- and sex-matched clinically healthy controls. Plasma samples from the participants were subjected to in-house enzyme-linked immunosorbent assay (ELISA) to determine their antibody levels.

**Results:**

Using ETBF total protein as coating antigen, 38/39 (97%) CRC cases and 36/39 (92%) controls showed anti-ETBF IgG above cut-off, while all (100%) CRC cases and 36/39 (92%) controls had anti-ETBF IgA levels above cut-off. With culture broth as coating antigen, all (100%) CRC cases and 37/39 (95%) controls had anti-ETBF IgG levels above cut-off. For anti-ETBF IgA, all (100%) cases and controls had levels above cut-off. Statistical analysis reveals no significant difference (*P* > 0.05) on the number of CRC cases and controls with IgG and IgA antibody levels above cut-off value. Also, there's no significant difference (*P* > 0.05) in the mean anti-ETBF antibody levels of cases who were at different tumor grade (well differentiated and moderately and poorly differentiated) and tumor stage (early and advanced).

**Conclusions:**

These results suggest that Filipino CRC cases and their clinically healthy matched controls exhibit antibody responses against ETBF.

Colorectal cancer (CRC) is an uncontrolled growth of abnormal cells in the colon or rectum. It begins as a benign tumor called polyp or adenoma on the epithelial cell mucosa of the colon or rectum and becomes cancer over a period of several years, approximately 10 years or more [1].

CRC is considered as one of the most prevalent and leading causes of cancer-related deaths in 2015 worldwide. Of all the cancer types, it ranks third in terms of incidence while second in terms of mortality for both sexes in 2018 [2]. In the Philippines, CRC is currently the third leading site of malignancy. The Philippine Society of Gastroenterology (PSG) identified CRC as the number 1 gastrointestinal cancer in the country with over 3,000 cases of CRC among Filipinos annually, out of which a death count of more than 2,000 has been recorded [3].

CRC development is being linked to dysbiosis, a distinct change or imbalance in the microbiota composition. Imbalance in colonic microbial population allows proliferation of pathogenic keystone species that can initiate induction of pro-inflammatory cytokines and disruption of epithelial tissue barrier function leading to carcinogenesis [4].

*Bacteroides fragilis* is commonly isolated in clinical infections despite comprising only approximately <1%–2% of the cultured fecal flora [5]. Enterotoxigenic *Bacteroides fragilis* (ETBF), the pathogenic strain of *B. fragilis*, is considered as one of the emerging etiologic agents of CRC due to its production of fragilysin or BFT – a 20 kDa zinc metalloprotease toxin that induces the cleavage of E-cadherin junction protein on epithelial cells, resulting in the disruption of E-cadherin and β-catenin linkage [6]. This event leads to augmented intestinal secretion, change in epithelial cell cytoskeletal structure [6], and the release of a portion of β-catenin [7]. Detachment of β-catenin from E-cadherin enhances cell signaling via β-catenin/Wnt pathway that may further trigger inflammation through nuclear localization and transcription of genes involved in tumor progression (c-*myc*) [7].

Several studies have been conducted in animal models and in CRC patients [8, 9] to investigate the connection of ETBF with CRC development. Molecular studies show that ETBF is significantly higher in CRC patients compared to healthy controls [9,9,11], suggesting its possible association with colorectal carcinogenesis. At present, there is no existing study on the potential role of ETBF with CRC development among Filipinos. In this study, we determined and compared the plasma anti-ETBF IgG and IgA levels of CRC patients with matched clinically healthy controls and determined whether anti-ETBF antibody levels of CRC patients are associated with tumor grade and tumor stage.

## Methods

**Figure 1.**
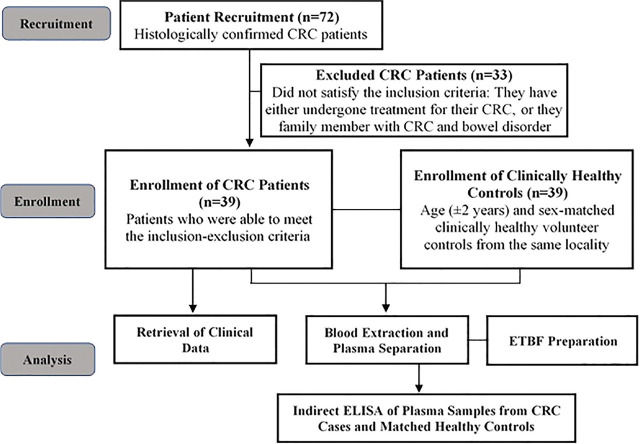
An overview of the method adopted in this study. CRC, colorectal cancer; ELISA, enzyme-linked immunosorbent assay; ETBF, enterotoxigenic *Bacteroides fragilis*.

The flowchart below provides a general overview of the procedures conducted in this study (**Figure 1**).

## Study samples

### Patient recruitment and sampling

Ethical clearances were obtained from the Research Ethics Review Committees (RERCs) of the hospital study sites – the Mariano Marcos Memorial Hospital and Medical Center in Ilocos Norte (MMMH-RERC-17-001) and the University of Santo Tomas Hospital (IRB-2016-11-191-IS-A1/CR2) in Manila. Written informed consent was obtained from all the participants.

In this case-control study, patients born to Filipino parents who were histologically confirmed CRC patients seen at University of Santo Tomas Hospital (USTH) and Mariano Marcos Memorial Hospital and Medical Center (MMMH-MC) between April 2018 and March 2019 were recruited and enrolled as cases. There were initially 72 potential CRC participants but only 39 were enrolled as cases since they were the only ones who have not yet undergone any form of treatment for their cancer during recruitment, their primary cancer is CRC, and they had no history of bowel disorder such as IBD, polyposis syndromes, and Lynch syndrome. Each of the cases was age- (±2 years) and sex-matched with physician-assessed clinically healthy controls from the same locality (**Figure 1**). The controls were physically assessed to be free from any form of malignancies. Clinical data of the cases were retrieved from histopathology reports and medical records. This prospective study followed the Strengthening the Reporting of Observational Studies in Epidemiology (STROBE) case-control reporting guidelines [12].

### Blood extraction

Blood collected in Ethylenediaminetetraacetic acid (EDTA) tubes from the enrolled participants was immediately centrifuged at 2,500 rpm for 15 min. Separated plasma samples were stored at −20°C until serologic analysis. All plasma samples were coded to avoid bias in the analysis and to maintain the confidentiality of the identity of the study participants.

### Enterotoxigenic *B. fragilis* preparation

The enterotoxigenic *B. fragilis* (ETBF 86-5443-2-2) culture used in this study was generously given by Dr. Cynthia Sears of John Hopkins University. Following the collection of 48 h ETBF culture grown anaerobically in brain heart infusion (BHI) agar, total protein was extracted using ReadyPrep^TM^ Protein Extraction Kit (Bio-Rad Laboratories) following the manufacturer's protocol with slight modification. The super-natant was collected, and protein concentration was determined using bicinchoninic acid (BCA) assay (QuantiPro^TM^ BCA Assay Kit, Sigma-Aldrich). For the culture broth, BHI culture broth was obtained from 48 h culture, which was centrifuged to separate the bacteria from the liquid media.

## Indirect enzyme-linked immunosorbent assay (ELISA) of plasma samples from CRC cases and matched healthy controls

### Assay optimization

The chessboard titration method was initially deployed to determine the optimum working conditions. ETBF lysate and culture broth were diluted with bicarbonate/carbonate coating buffer (pH 9.6) to concentrations of 80 μg/mL, 50 μg/mL, 25 μg/mL, 10 μg/mL, and 5 μg/mL. Pooled plasma samples were diluted to 1:50, 1:100, 1:500, and 1:1,000 and the horseradish peroxidase (HRP)-conjugated anti-IgG and anti-IgA with a concentration of 1.2 mg/mL (Abcam) were diluted to 1:500 and 1:1,000. ELISA of the pooled samples from CRC patients and healthy controls was performed using the ETBF lysate coating concentration (micrograms per milliliter), plasma sample dilution, and HRP-conjugated secondary antibody dilution, which were simultaneously tested. The optimum working conditions were decided based on the combined lowest concentrations of the different components that gave the strongest signal versus low background.

### ELISA assay set-up

Optimized indirect ELISA conditions identified after deployment of the chessboard titration method were used to determine the IgG and IgA antibody levels of CRC patients and their case-matched clinically healthy controls against ETBF. The 96-well plates were coated with 50 μL of 5 μg/mL ETBF lysate, incubated overnight, and blocked with bovine serum albumin (BSA) for 1 h. This was followed by addition of 50 μL of 1:100 diluted plasma of CRC patients and their case-matched controls. Following 1 h of incubation, HRP-conjugated goat anti-human IgG or IgA antibodies (50 μL; 1:1,000) (Abcam) were dispensed in each well and incubated for 1 h. Visualization was performed by adding 50 μL of tetramethyl benzidine (TMB) (TMB Substrate Set, BioLegend), followed by reading the absorbance at 450 nm (OD_450 nm_) (SPECTROstar Nano, BMG Labtech). The same procedure was performed using the ETBF culture broth.

### Calculations of cut-off values (COVs)

The COVs of anti-ETBF IgG or IgA antibody levels of the control samples were used in determining positive reactions among the CRC cases. The COVs were computed based on the formula: $\bar{\text{x}}\;+\;\text{SD}\;\text{t}\sqrt{1+\left(1/n\right)}$ [13], where SD is standard deviation.

### Statistical analysis used in the study

Samples with absorbance values above the respective COV were considered reactive or positive while all values below COV were considered non-reactive or to have negative reactivity with the ETBF lysate. The plasma reactivity of cases and controls in terms of anti-ETBF IgG and IgA antibody titers was also compared using an independent *t* test. The association of antibody levels with tumor grade and tumor stage was ascertained using an independent sample *t* test, in which cases with missing data were not included. Stata 14 was used to analyze the data and *P* < 0.05 was considered significant.

## Results

### Study samples

A total of 39 histologically confirmed preoperative/pretreatment CRC cases and 39 age- and sex-matched clinically assessed healthy controls seen at the University of Santo Tomas Hospital (Manila) and Mariano Marcos Memorial Hospital and Medical Center (Ilocos Norte, Philippines) between April 2018 and March 2019 were enrolled in this study.

The majority of the CRC cases were males (n = 22, 56%) who were aged more than 50 years with moderately and poorly differentiated (n = 17, 44%) tumors, and were in an advanced stage (III/IV) of the disease (n = 21, 54%) (**Table 1**).

**Table 1.** Demographic and clinical characteristics of CRC cases and controls

**Characteristics**	**Cases (n = 39)**	**Controls (n = 39)**	*P* value^*^
Sex			
Male	22 (56%)	22 (56%)	
Female	17 (44%)	17 (44%)	
Age at initial diagnosis (years)			
<50	11 (28%)	10 (26%)	0.49
≥50	28 (72%)	29 (74%)	0.43
Mean age at initial diagnosis (years ± SD)	58 ± 12		
Median age at initial diagnosis	61		
Tumor grade			
G1 (well differentiated)	11 (28%)		
G2 & G3 (moderately and poorly differentiated)	17 (44%)		
No information available	11 (28%)		
Tumor stage			
T1 & T2 (early)	8 (20%)		
T3 & T4 (advanced)	21 (54%)		
No information available	10 (26%)		

* Independent *t* test with equal variances.

CRC, colorectal cancer.

## In-house ELISA of plasma samples

### The optimization assay

**Figure 2.**
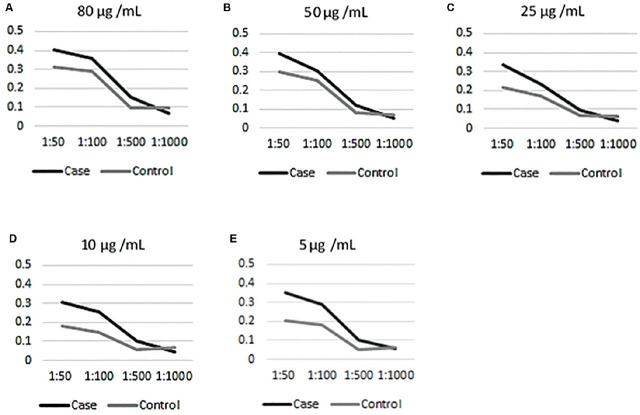
Checkerboard titration results at 1:1,000 secondary antibody dilution: X-axis = primary antibody (pooled plasma samples) concentration; Y-axis = anti-ETBF IgG antibody level absorbance at OD_450_ nm. (**A**) 80 μg/mL coating antigen; (**B**) 50 μg/mL coating antigen; (**C**) 25 μg/mL coating antigen; (**D**) 10 μg/mL coating antigen; (**E**) 5 μg/mL coating antigen. ETBF, enterotoxigenic *Bacteroides fragilis*.

The optimum working conditions were based on the lowest concentrations of the different components that gave the strongest signal versus low background. In this study, the optimum conditions were 5 μg/mL for the coating protein, 1:100 plasma dilution, and 1:1,000 detection antibody dilution (**Figure 2E**).

### ELISA assay set-up

COVs for ETBF lysate were 0.074 and 0.077 for IgG and IgA antibodies, respectively. Using culture broths, COVs for IgG and IgA antibodies were ascertained at 0.079 and 0.074, respectively.

**Figure 3.**
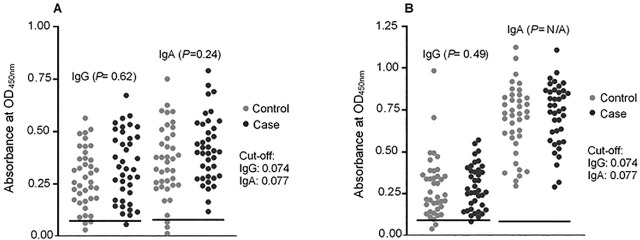
Comparison of optical density (OD) values (nanometer) of anti-ETBF IgG and anti-ETBF IgA levels between CRC patients and healthy individuals (cases, n = 39; controls, n = 39). (**A**) Bacterial lysate as coating antigen; (**B**) culture broth as coating antigen. CRC, colorectal cancer; ETBF, enterotoxigenic *Bacteroides fragilis*.

Using ETBF lysate as coating antigen, it was ascertained that 38 (38/39, 97%) of the CRC cases had anti-ETBF IgG antibodies above the cut-off, while all (39/39, 100%) of the cases had anti-ETBF IgA antibodies above the cut-off. Likewise, all (39/39, 100%) cases tested positive for anti-ETBF IgG and IgA antibodies to ETBF culture broths as coating antigen. For the clinically matched controls, 36/39 or 92% had anti-ETBF IgG and IgA antibodies above the cut-off when bacterial lysates were used. All the cases and controls (39/39, 100%) were positive for anti-ETBF IgA antibodies when ETBF culture broth was used as coating antigen. Statistical analysis showed no significant difference on the number of CRC cases who tested positive for anti-ETBF IgG (lysate: *P* = 0.615; broth: *P* = 0.494) and IgA (*P* = 0.240) antibodies compared to their matched controls in both bacterial lysates and culture broths (**Figure 3**).

### Association of tumor grade and tumor stage with anti-ETBF antibody levels using bacterial lysates

**Figure 4.**
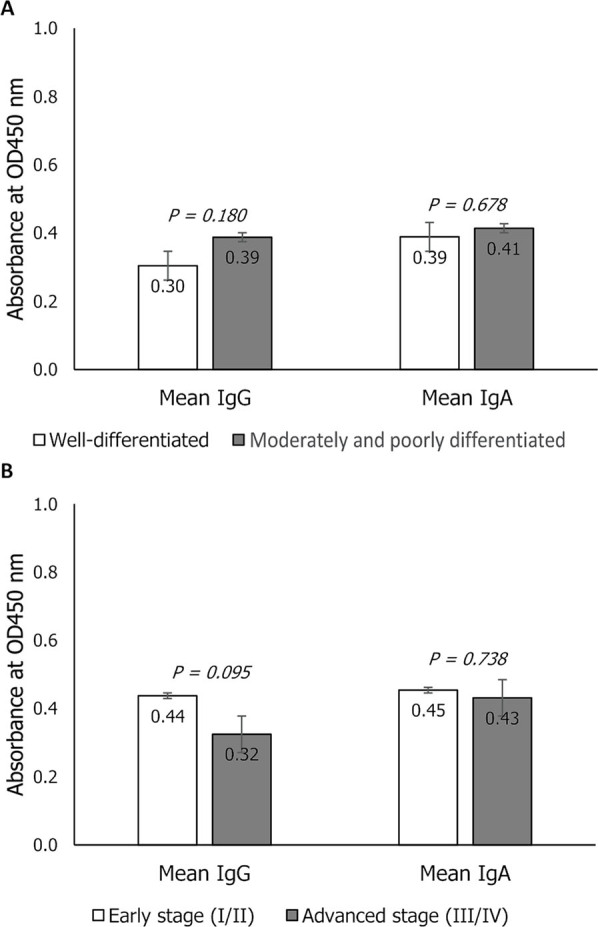
Comparison of the mean OD values (nanometer) of the anti-ETBF IgG and IgA antibody levels of CRC patients when grouped by (**A**) tumor grade and (**B**) tumor stage using bacterial lysate as coating antigen. CRC, colorectal cancer; ETBF, enterotoxigenic *Bacteroides fragilis*.

CRC cases with well-differentiated tumors (11/28, 39%) and moderately to poorly differentiated tumors (17/28, 61%) had higher IgA than IgG levels against ETBF. Similarly, cases at the early (8/29, 28%) and advanced tumor stage (21/29, 72%) had greater anti-ETBF IgA than IgG levels. When categories within tumor grade were analyzed, the result showed that the mean IgG and IgA levels of those with moderately and poorly differentiated tumor were not significantly higher than those of the cases with well-differentiated tumor (IgG: 0.3876 ± 0.1632 vs. 0.3042 ± 0.1556, *P* = 0.180; IgA: 0.4139 ± 0.1719 vs. 0.3887 ± 0.1410, *P* = 0.678). There were no significant differences in the antibody levels observed in CRC cases at the early and advanced stages of malignancy (IgG: 0.4374 ± 0.1246 vs. 0.3244 ± 0.1667, *P* = 0.095; IgA: 0.4538 ± 0.1665 vs. 0.4313 ± 0.1576, *P* = 0.738) (**Figure 4**).

**Figure 5.**
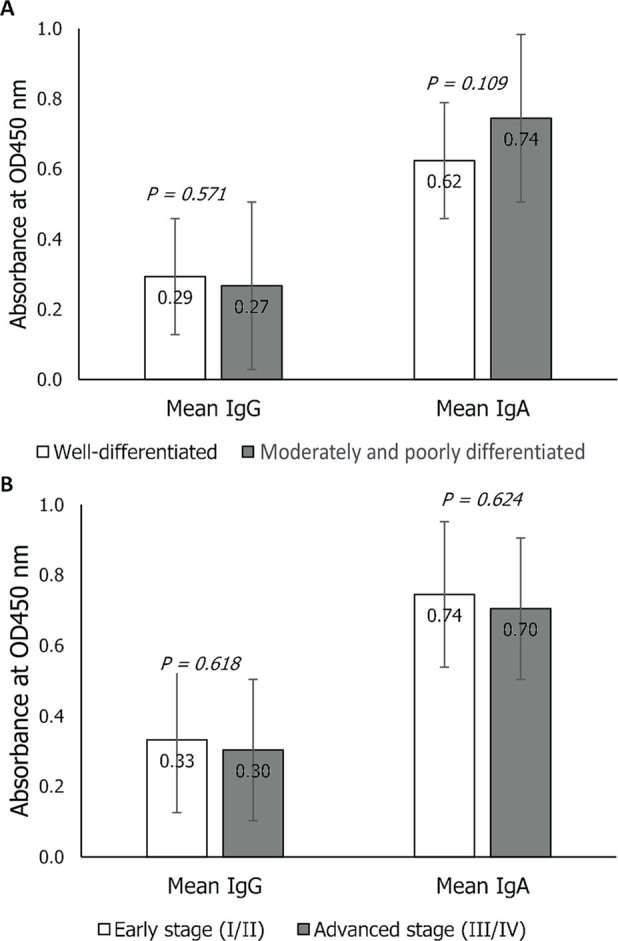
Comparison of the mean OD values (nanometer) of the anti-ETBF IgG and IgA antibody levels of CRC patients when grouped by tumor grade (**A**) and tumor stage (**B**) using bacterial culture broth as coating antigen. CRC, colorectal cancer; ETBF, enterotoxigenic *Bacteroides fragilis*.

### Association of tumor grade and tumor stage with anti-ETBF antibody levels using culture broth

The mean IgA antibody levels of cases, whether having well, moderately, or poorly differentiated tumor, at the early or advanced tumor stage, were higher than their IgG levels against ETBF. *T* test analysis showed that the mean anti-ETBF IgG and IgA levels of CRC cases with well-differentiated tumors were not significantly different from the mean antibody levels of the cases with moderately and poorly differentiated tumors (IgG: 0.2932 ± 0.1191 vs. 0.2671 ± 0.1200, *P* = 0.571; IgA: 0.6238 ± 0.1907 vs. 0.7448 ± 0.1938, *P* = 0.109). Likewise, the mean anti-ETBF IgG and IgA levels of early-stage CRC were not significantly higher than those of advanced stage CRC (IgG: 0.3320 ± 0.0760 vs. 0.3034 ± 0.1191, *P* = 0.618; IgA: 0.7448 ± 0.1930 vs. 0.7044 ± 0.1965, *P* = 0.624) (**Figure 5**).

## Discussion

This study shows that anti-ETBF IgG and IgA levels produced by CRC patients are not significantly different from those of matched clinically healthy controls. In previous studies, significantly higher ETBF colonization in CRC cases than in healthy individuals was reported [10–11]. These observations might indicate secretion of greater amount of the toxin in the colonic mucosa. Since the toxin of ETBF – *Bacteroides fragilis* Toxin (BFT) is proposed to modulate mucosal immune response [14], we expected that antibody levels against ETBF in CRC patients would be significantly higher than in the control group. In contrast, the present study shows comparable antibody titers between CRC cases and healthy controls. Our data may suggest that ETBF has induced immunologic response in CRC cases but might not be strong enough to be non-comparable with the clinically healthy controls. While results of this study show no significant difference on the number of histologically confirmed CRC cases positive for anti-ETBF IgG and IgA antibodies, it cannot be assumed yet that this organism has no oncogenic potential.

The present study agrees with the results of another study [15], wherein the anti-tcdB IgG and IgA levels were not associated with either tumor stage or tumor grade. Based on the “driver-passenger” theory [16], ETBF was proposed to be one of the “driver bacteria” that initiate colorectal carcinogenesis. “Driver bacteria” induce CRC by promoting epithelial cell DNA damage and tumorigenesis, which then encourage “passenger bacteria” with tumor-promoting properties to proliferate given their growth advantage in the tumoral environment. Supposing that this is true, ETBF should be enriched in individuals who are in an early stage of their malignancy. Higher ETBF colonization might mean higher antibody levels against the bacterium. The result of our study supports the said theory, although several studies show higher occurrence of ETBF in tissue samples of late-stage (III/IV) CRC patients compared to those in the early stage (I/II) [9, 11].

The presence of opportunistic bacteria and their antigens in the colonic mucosa may induce disruption of the gut epithelial barrier, thereby leading to chronic inflammation [6]. A long period of exposure to these bacteria can induce humoral immune response, which results in the production of antibodies such as IgG that confer long-term protection. However, antibody titers may decline over time, such as in hepatitis B virus antibody [17]. The possibility of this happening in CRC patients has been observed.

Serologic testing for ETBF antibodies is simpler and can potentially be used for diagnosis of diseases such as those “microbially-induced” cancers. However, this test may possibly produce false positive or false negative results and can no longer be reliable once production of antibody specific to the antigen we are interested in has lapsed. Moreover, since we used plasma samples to detect anti-ETBF IgA antibody, results may not show the actual concentration of the antibody since IgA is predominantly secreted and present in mucosal surfaces. The sensitivity of the assay might have also been sacrificed due to the use of crude protein lysate as coating antigen instead of specific BFT (fragilysin). The use of crude antigen or antibody preparations may result in a low effective antigen/antibody concentration, which can be outcompeted by contaminating proteins, thus leading to very low signal [17]. Also, the observed antibody titers may not show the actual and specific antibody levels against fragilysin. These things together with the small number of samples analyzed pose a limitation in our study.

Despite the increasing trend of CRC incidence and mortality in the Philippines [2], limited studies have been conducted to investigate its etiology. At present, available studies include: the analysis of *Clostridioides difficile's* anti-tcdB antibody, which revealed a significantly higher number of preoperative CRC cases who were positive for the anti-tcdB IgG and IgA antibodies compared with their healthy controls [14]; and a study on the Fap2 protein of *Fusobacterium nucleatum*, which showed that most of the CRC cases were reactive to the synthetic mimotope of Fap2 protein [18].

Up to this date, the prime driver of colorectal carcinogenesis has not yet been identified. The etiology of CRC is multifactorial and has been linked with genetic and environmental factors such as genetic mutations, gut microbiota, diet, and geographic location. Family history and age are also important risk factors aside from ethnicity and lifestyle [19]. In addition, it has been suggested that the genetic makeup of an individual could possibly influence bacterial colonization patterns and the subsequent pathology [16]. Environmental factors and host genetic susceptibility influencing the microbiome–host immune system interaction may lead to immune-mediated diseases.

## Conclusion

Our study suggests that Filipino CRC cases exhibit antibody response against Enterotoxigenic *B. fragilis*. ETBF induces immune response in both CRC cases and healthy individuals, although anti-ETBF IgG and IgA levels do not distinguish those who have colorectal malignancy from healthy individuals. This study, despite the small number of enrolled cases, contributes to the existing limited data on the role of gut microbiome, especially enterotoxigenic *B. fragilis*, in CRC development among Filipinos.
